# Une cholécystite sur moignon

**DOI:** 10.11604/pamj.2015.22.241.8217

**Published:** 2015-11-13

**Authors:** Ammar Mahmoudi, Khadija Zouari

**Affiliations:** 1 Service de Chirurgie Générale et Digestive, CHU Fattouma Bourguiba de Monastir, Tunisie

**Keywords:** Cholécystite lithiasique, cholécystectomie sub-totale, moignon vésiculaire, tomodensitométrie, cholangio-IRM, chirurgie, gallstone cholecystitis, subtotal cholecystectomy, gallbladder stump, CT scan, MRI cholangiography, surgery

## Image en medicine

Une cholécystectomie subtotale expose au risque de cholécystite sur moignon, bien qu'elle constitue une option sûre face à une inflammation sévère du triangle de Calot permettant de réduire le risque de plaie biliaire. Malgré une histoire parfois typique, le diagnostic est souvent retardé en raison d'une faible suspicion. Par conséquent les cliniciens devraient évoquer ce diagnostic. Une fois le diagnostic confirmé, il convient de réaliser une ré-intervention pour exciser le moignon. Nous rapportons le cas d'un patient agé de 43 ans opéré par laparotomie sous-costale droite pour un pyo-cholécyste lithiasique. Il a été réalisé une cholécystectomie. L'examen anatomo-pathologique avait conclu à une cholécystite aiguë avec péri-cholécystite. Deux ans après, le patient présentait un syndrome douloureux et fébrile de l'hypocondre droit depuis trois jours et a été traité à tort pour pneumopathie droite avec une radiographie thoracique normale. A l'examen, le patient était fébrile à 38,5°c, et il existait une défense à la palpation de l'hypocondre droit. Il existait un syndrome inflammatoire biologique. L'échographie était non contributive. La tomodensitométrie (A) avait montré un aspect d'une cholécystite sur moignon avec une infiltration importante tout autour. L'IRM abdominale (B,C) avait montré une néo-cavité au niveau du lit vésiculaire d'environ 3,5x1,5 cm contenant une concrétion lithiasique ovalaire de 15 mm de grand axe qui se continue avec le canal cystique sans dilatation des voies biliaires. Sous couverture antibiotique, la reprise de la sous-costale avait permis, difficilement, l'exérèse du moignon et une cholangiographie per-opératoire montrant une vacuité des voies biliaires. Les suites opératoires étaient simples.

**Figure 1:**
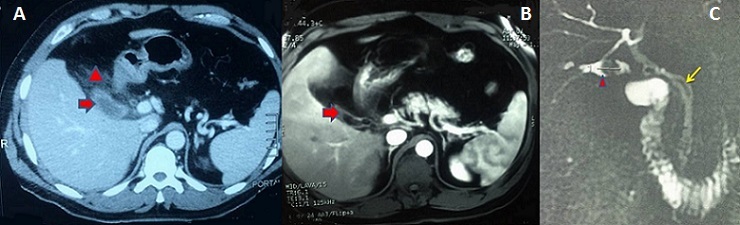
(A) tomodensitométrie abdominale en coupe axiale: Présence en sous-hépatique d'un moignon vésiculaire à paroi épaissie (flèche) associé à une importante infiltration tout autour (tête de flèche); (B) IRM abdominale en coupe axiale: présence d'une néo-cavité au niveau du lit vésiculaire d'environ 3,5 x 1,5 cm contenant une concrétion lithiasique ovalaire (flèche); (C) cholangio-IRM: Présence d'un moignon vésiculaire, à paroi épaissie, d'environ 3,5 x 1,5 cm contenant une concrétion lithiasique ovalaire de 15 mm de grand axe (tête de flèche). Le moignon se continue avec le canal cystique (flèche) qui est long s'implantant à gauche de la voie biliaire principale. Pas de dilatation des voies biliaires intra et extra-hépatiques

